# Triple Contrast CT Method Enables Simultaneous Evaluation of Articular Cartilage Composition and Segmentation

**DOI:** 10.1007/s10439-019-02362-6

**Published:** 2019-10-01

**Authors:** Miitu K. M. Honkanen, Annina E. A. Saukko, Mikael J. Turunen, Wujun Xu, Goran Lovric, Juuso T. J. Honkanen, Mark W. Grinstaff, Vesa-Pekka Lehto, Juha Töyräs

**Affiliations:** 1 Department of Applied Physics, University of Eastern Finland, P.O. Box 1627, 70211 Kuopio, Finland; 2 Diagnostic Imaging Center, Kuopio University Hospital, Kuopio, Finland; 3 Department of Medical Physics, Turku University Hospital, Turku, Finland; 4 SIB-labs, University of Eastern Finland, Kuopio, Finland; 5 Centre d’Imagerie BioMédicale, École Polytechnique Fédérale de Lausanne, Lausanne, Switzerland; 6 Swiss Light Source, Paul Scherrer Institute, Villigen, Switzerland; 7 Center of Oncology, Kuopio University Hospital, Kuopio, Finland; 8 Departments of Biomedical Engineering, Chemistry, and Medicine, Boston University, Boston, MA USA; 9 School of Information Technology and Electrical Engineering, The University of Queensland, Brisbane, Australia

**Keywords:** Triple contrast agent, Dual contrast agent, Computed tomography, Contrast-enhanced computed tomography, Post-traumatic osteoarthritis, Synchrotron microCT

## Abstract

Early degenerative changes of articular cartilage are detected using contrast-enhanced computed tomography (CT) with a cationic contrast agent (CA). However, cationic CA diffusion into degenerated cartilage decreases with proteoglycan depletion and increases with elevated water content, thus hampering tissue evaluation at early diffusion time points. Furthermore, the contrast at synovial fluid-cartilage interface diminishes as a function of diffusion time hindering accurate cartilage segmentation. For the first time, we employ quantitative dual-energy CT (QDECT) imaging utilizing a mixture of three CAs (cationic CA4+ and non-ionic gadoteridol which are sensitive to proteoglycan and water contents, respectively, and bismuth nanoparticles which highlight the cartilage surface) to simultaneously segment the articulating surfaces and determine of the cartilage condition. Intact healthy, proteoglycan-depleted, and mechanically injured bovine cartilage samples (*n *= 27) were halved and imaged with synchrotron microCT 2-h post immersion in triple CA or in dual CA (CA4+ and gadoteridol). CA4+ and gadoteridol partitions were determined using QDECT, and pairwise evaluation of these partitions was conducted for samples immersed in dual and triple CAs. In conclusion, the triple CA method is sensitive to proteoglycan depletion while maintaining sufficient contrast at the articular surface to enable detection of cartilage lesions caused by mechanical impact.

## Introduction

Osteoarthritis (OA) arises due to prolonged use, over use, or injury of an articulating joint with a breakdown of cartilage and sclerosis of subchondral bone causing pain, joint stiffness, swelling, and mobility loss.^1^,^4^,^32^,^35^ Post-traumatic osteoarthritis (PTOA) is one form of OA caused by an acute injury of a knee joint resulting from a sport accident, a fall, or any other source of physical trauma, and it primarily affects younger individuals.^1^ However, by identifying OA early on, the progression of the damage or injury can be slowed down or even prevented using surgical and pharmaceutical interventions.^1^,^41^ Thus, methods for early diagnosis of cartilage injuries are needed. Magnetic resonance imaging (MRI) is an important tool in diagnosis of cartilage degeneration and PTOA. It is sensitive to the water content in cartilage tissue, the 3D architecture of the collagen network, and also to proteoglycan (PG) content.^27^,^30^ However, MRI suffers from its relatively long *in vivo* imaging acquisition times, high costs, and being unsuitable for imaging patients with implanted medical devices that are incompatible with MRI.^26^,^30^

Contrast-enhanced computed tomography (CECT) allows imaging with high-resolution, short scan times and, thus, with less motion artifacts at approximately half the cost of MRI, providing an alternative imaging method to evaluate the cartilage condition. Even though CECT exploits ionizing radiation for extremity imaging, the doses involved in clinical cone-beam CT (CBCT) instruments are low with effective doses below 50 *µ*Sv.^20^ CECT utilizes contrast agents that enhance the contrast at synovial fluid-cartilage interface where the natural contrast is nearly non-existent due to similar X-ray absorptions. In addition to providing the contrast between cartilage and synovial fluid, contrast agents reveal degenerative changes in cartilage.^16^,^17^,^34^,^42^,^47^,^48^,^53^ The uptake and diffused partition of contrast agents are altered in degenerated cartilage tissue due to degeneration-related changes including (1) decreased cartilage fixed charge density (FCD) *via* loss of PGs, (2) increased water content, and (3) disruption of the superficial collagen network.^7^,^28^ Therefore, CECT could be used along with MRI to detect internal articular cartilage and meniscus^11^,^24^ degeneration after acute injury. In addition, CECT allows simultaneous assessment of the bony structures of the joint.^39^,^55^

Anionic or non-ionic contrast agents are traditionally used in CECT imaging but a recently introduced cationic contrast agent (CA4+) shows superior sensitivity at diffusion equilibrium to detect tissue PG content.^2^,^24^,^25^ Despite this, the widespread use of CA4+ is hindered due to suboptimal performance at imaging times during early diffusion (0–2 h from contrast agent administration).^18^,^22^ The distribution of cationic contrast agents with articular cartilage is proportional to PG content as the negative fixed charge carried by PG molecules attracts the positively charged contrast agents. However, especially in the early stage of diffusion, the diffusion of cationic contrast agent is also controlled by degeneration-related factors having opposite effects; the loss of PGs decreases the diffusion of cationic contrast agent while increase in water content and decrease in steric hindrance (i.e., physical diffusion barrier of the tissue caused by collagen network architecture and PGs in the matrix) increase the diffusion. This shortcoming can be overcome using a quantitative dual-energy CT technique (QDECT).^5^

QDECT utilizes a mixture of a cationic, iodinated contrast agent (e.g., CA4+)^2^,^16^ and a non-ionic, gadolinium-based contrast agent (e.g., gadoteridol). As mentioned above, CA4+ possesses a high affinity for PGs. Gadoteridol, on the other hand, as an uncharged molecule distributes into cartilage according to water content and steric hindrance. Thus, by normalizing (e.g., dividing) the cationic contrast agent partition in the cartilage with that of the non-ionic contrast agent, the effects of water content and steric hindrance on the diffusion of the cationic contrast agent can be limited.^5^,^12^,^46^ Simultaneous determination of the uptake of the both contrast agents within cartilage requires imaging with two X-ray energies. In QDECT, these energies are selected based on element specific absorption *k*-edge energies of iodine (33.2 keV) and gadolinium (50.3 keV).

Current CECT of a knee joint requires two subsequent CT scans acquired immediately (arthrography) and 30 min to 2 h (delayed arthrography) after the intra-articular injection of contrast agent.^22^,^23^,^39^ The first scan enables accurate segmentation of articulating surface and lesions while the latter scan provides information on internal changes in cartilage tissue and properties related to the initiation of PTOA by examining the diffusion of the contrast agent in the tissue. The two scans are required since the segmentation of articulating surface and delineation of lesions is difficult from the latter scan owing to diffusion-related loss of image contrast at the synovial fluid-cartilage interface. In addition, no interpretation of the cartilage tissue properties is made from the first scan as the contrast agent has not had sufficient time to diffuse into cartilage. Acquiring two images is logistically burdensome and doubles the radiation dose to a patient. Our recent findings have shown that bismuth nanoparticles (BiNP) can be utilized to overcome this problem of requiring two CT scans.^45^ Due to their size, BiNPs are too large to diffuse into the cartilage tissue, and, thus, remain at the synovial space.^45^ Using the QDECT with BiNPs and ioxaglate, only the delayed arthrography is required since both the segmentation and evaluation of the cartilage condition can be done simultaneously.

**Figure 1:**
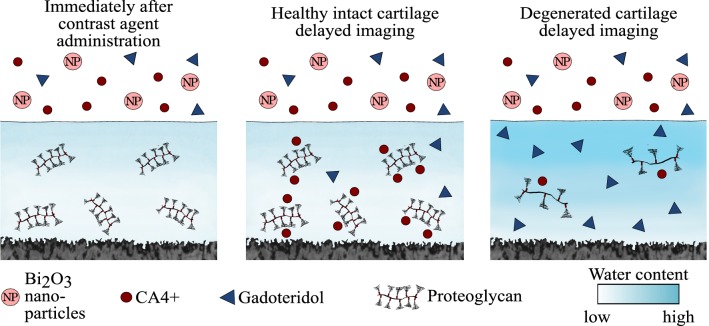
The uptake of cationic contrast agent (CA4+) is proportional to the fixed charge density conferred by proteoglycans (PGs). Healthy cartilage has a high PG content, and thus the uptake of cationic contrast agent (CA4+) is also high. In degenerated cartilage the uptake of cationic contrast agent into cartilage matrix is limited due to decreased PG content. On the other hand, as the tissue degenerates, the tissue water content increases and steric hindrance decreases allowing more contrast agent molecules (both CA4+ and gadoteridol) to penetrate the tissue. Bismuth nanoparticles (average diameter of 194 nm) are too large to be able to diffuse into either healthy or degenerated cartilage, thus maintaining the contrast at the articulating surface at all diffusion time points.

In this study, we introduce for the first time a triple contrast agent composed of three contrast agents: (1) cationic, iodine-based CA4+, (2) non-ionic, gadolinium-based gadoteridol, and (3) BiNP suspension. Using the triple contrast agent, we hypothesize that the PG and interstitial water contents in articular cartilage will be simultaneously and quantitatively assessed based on CA4+ and gadoteridol distributions within tissue (Fig. 1). Further, we hypothesize that accurate segmentation of the articulating surface from the delayed CT arthrography images will be facile as BiNPs maintain good contrast between the articulating surface and immersion bath. To address these hypotheses, intact healthy, proteoglycan-depleted, and mechanically injured cartilage samples are evaluated using synchrotron microCT as it provides fast tomographic imaging with monochromatic X-ray spectra and high resolution.^51^

## Materials and Methods

**Figure 2:**
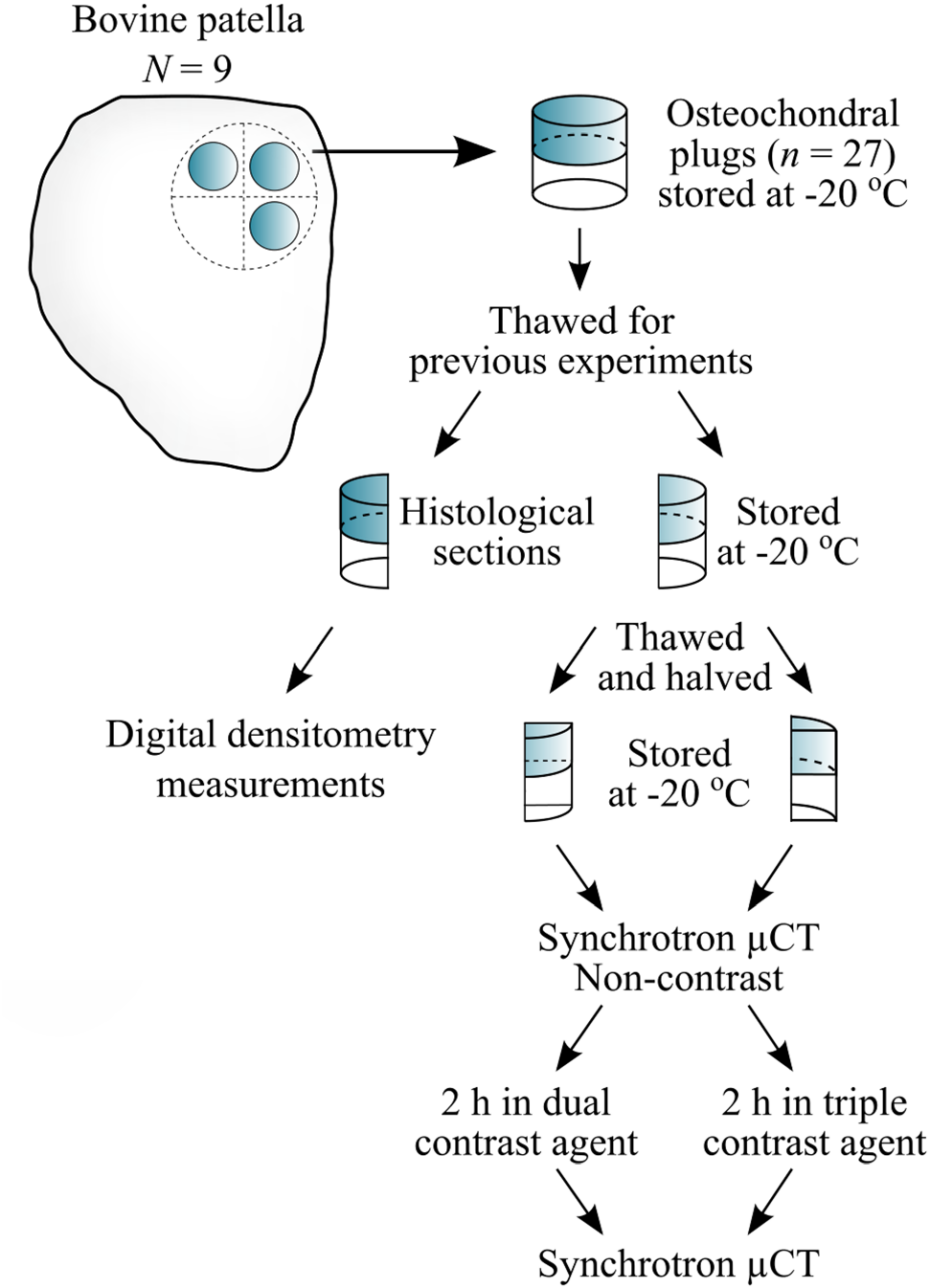
Workflow of the sample preparation and processing protocol. The samples were thawed for previous experiments described in Saukko *et al.*^45^ Bovine osteochondral samples were immersed in dual (mixture of CA4+ and gadoteridol) or in triple (mixture of CA4+, gadoteridol and bismuth nanoparticles) contrast agents for 2 h before the synchrotron microCT (µCT) measurements.

Intact bovine patellae (*N* = 9) were dissected from skeletally mature stifle joints obtained from a local butcher (Savo-Karjalan liha Oy, Finland) and stored in − 20 °C wrapped in phosphate buffered saline (PBS) soaked gauze in a zip lock bag until sample preparation (a detailed flowchart of the sampling and experiments are presented in Fig. 2). Prior to the sample preparation, the patellae were thawed in a bath of PBS in room temperature, and three adjacent osteochondral plugs (*n* = 27, *d* = 7 mm) were extracted from the upper lateral quadrant of the patellae. Osteochondral plugs were trimmed to include 1 mm of bone underneath the cartilage.^8^ The osteochondral plugs were divided into three sample groups: (1) intact, (2) PG-depleted, and (3) mechanically injured. The latter two served as models of cartilage degeneration and acute injury, respectively. Early-stage OA involving PG-loss can be mimicked by treating the cartilage with proteolytic enzymes.^3^,^6^,^10^,^29^,^38^,^44^,^45^ Nearly complete PG-depletion in sample group 2 was achieved by immersing the samples in PBS supplemented with trypsin (0.5 mg/mL, Sigma-Aldrich, MO, USA) for 15 h at 37.5 °C, 5% CO_2_ atmosphere in an incubator.^38^,^45^ Subsequently, trypsin-treated samples were immersed in PBS for 2 h at 7 °C to suppress the degradation process. To mimic disruption of the cartilage surface (related to PTOA) and cartilage lesion after the physical trauma caused by impact, a mechanical injury was induced to cartilage.^15^ This damage was accomplished using a custom-made drop tower with a stainless-steel impactor (500 g) having a flat polished face.^21^,^45^ The impactor was dropped on the sample from the height of 20 cm^21^,^45^ creating cracks with varying depth and size on the articular surface. After the impact, the impactor was immediately lifted from the articular surface, and the samples were allowed to recover in PBS for 2 h to prevent creep deformation. Then, the samples were imaged with a double contrast agent (mixture of ioxaglate and BiNPs) as described in our previous study,^45^ and the contrast agent was subsequently washed out (by immersing the samples in PBS for 2 h) and the samples were frozen (− 20 °C) in a zip lock bag as immersed in fresh PBS. Prior to the measurements for this study, the samples were thawed and halved (Fig. 2). The first half was used in digital densitometry (DD) measurements to determine the optical density of Safranin-O stained sections (i.e., the PG content) of the sample while the second half was halved again for pairwise evaluation of dual and triple contrast agents. The sides and the bottom of the quarter osteochondral samples intended for synchrotron microCT imaging were carefully sealed with cyanoacrylate (Loctite, Henkel Norden AB, Dusseldorf, Germany) and stored at − 20 °C until the measurements. The quarter samples were measured at synchrotron microCT beamline (X02DA TOMCAT beamline of the Swiss Light Source, Paul Scherrer Institut (PSI), Villigen, Switzerland).^50^

BiNPs were prepared by ball-milling (Planetary Micro Mill, Pulverisette 7, Fritsch GmbH, Germany) bismuth(III) oxide powder in two standard milling bowls each containing 10 g of powder, 70 g of 1 mm milling balls, and 40 mL of distilled water. The bismuth oxide powder was milled in 10 min cycles at a speed of 700 rpm with a cooling time of 15 min in between each milling round resulting in a total milling time of 2 h. After milling, 0.5 kDa PEG-silane (2-[Methoxy(polyethyleneoxy)9-12propyl]trimethoxysilane, tech-90, Fluorochem, Old Glossop, UK) was added to the milled NP solution to improve the stability with the mass ratio of PEG-silane to BiNP being 1:6. Next, the mixture was heated up to 260 °C and maintained at a constant temperature for 2.5 h in a protective N_2_ atmosphere. Finally, to remove remaining unreacted chemicals, the solution was washed with ethanol three times *via* centrifuge separation (10,000 rpm, 10 min) and ultrasound re-dispersion. The obtained PEG-coated BiNPs were stored in ethanol until the synchrotron microCT measurements. The BiNPs possess a mean diameter of 194 nm and a surface charge of − 3.5 mV (Zetasizer Nano ZS, Malvern Instruments Ltd., Malvern, UK). Prior to measurements, the BiNPs were separated from the solution by centrifuging the BiNP-ethanol solution at 10,000 rpm for 5 min. Then, the NPs were dispersed in distilled water using ultrasound. Subsequently, the centrifuging and re-dispersion process was repeated similarly to ensure that all ethanol was completely washed out from the solution. After the preparation, the NPs were immediately mixed with the other contrast agents and the samples were immersed in the mixture.

**Figure 3:**
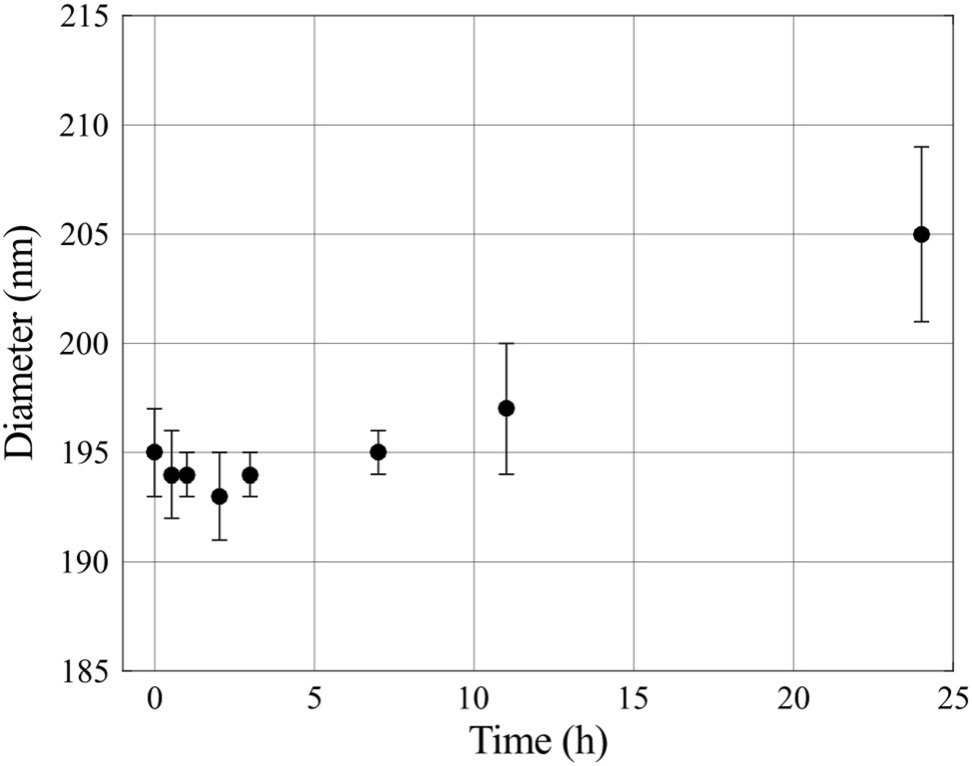
The stability of the bismuth nanoparticles was determined by measuring the particle diameter after the CA4+ and gadoteridol were added to the triple contrast agent mixture. The particle diameter change was not significant within 24 h according to the statistical analysis with the One-way ANOVA model (*p* > 0.05), as compared with the original particle diameter.

Two contrast agent mixtures were prepared; a dual contrast agent and a triple contrast agent. The isotonic (~ 308 mOsm/kg) dual contrast agent composed of a mixture of iodinated cationic contrast agent (CA4+, *q *= + 4, *M *= 1499.88 g/mol) and gadolinium-based, electrically neutral contrast agent (gadoteridol, *q *= 0, *M *= 558.69 g/mol, ProHance™, Bracco Diagnostic Inc., Monroe Twp., NJ, USA) was diluted in PBS. In the dual contrast agent, the iodine (I) concentration was 5 mg I/mL and gadolinium (Gd) 10 mg Gd/mL. Moreover, the solution was supplemented with proteolytic inhibitors [5 mM ethylenediaminetetraacetic acid (EDTA, VWR International, France) and 5 mM benzamidine hydrochloride hydrate (Sigma-Aldrich Inc., St Louis, MO, USA)] to suppress general protein degradation in cartilage tissue. Triple contrast agent was otherwise similar to dual contrast agent but BiNPs, with the concentration of 10 mg/mL of BiNPs, were added to the solution. The stability of the BiNPs in the triple contrast agent was analyzed *via* monitoring the particle diameter change during different time periods. The results (Fig. 3) indicate that the BiNPs are stable in the mixed triple contrast agent for more than 24 h.

Synchrotron-based microCT imaging was performed with a third-generation synchrotron-based X-ray source. MicroCT images were acquired by combining 1:1 magnifying visible light optics microscope (Optique Peter, France), a 300 *µ*m thick scintillator (LuAg, CRYTUR spol.s.r.o., Czech Republic), and a scientific complementary metal–oxide–semiconductor (sCMOS) detector (pco.Edge 5.5, PCO AG, Germany). Two monochromatic X-ray energies of 32 and 34 keV from both sides of iodine *k*-edge (33.2 keV) were selected to maximize the difference in the mass attenuation coefficients of iodine (CA4+). A double-multilayer monochromator with a spectral bandwidth of about 2–3% was used. Imaging geometry resulted in an isometric voxel size of 6.5 × 6.5 × 6.5 *µ*m^3^ and a field of view (FOV) of 16.6 × 3.5 mm^2^. The radiation exposure was minimized by applying an off-beam alignment system.^33^

**Figure 4:**
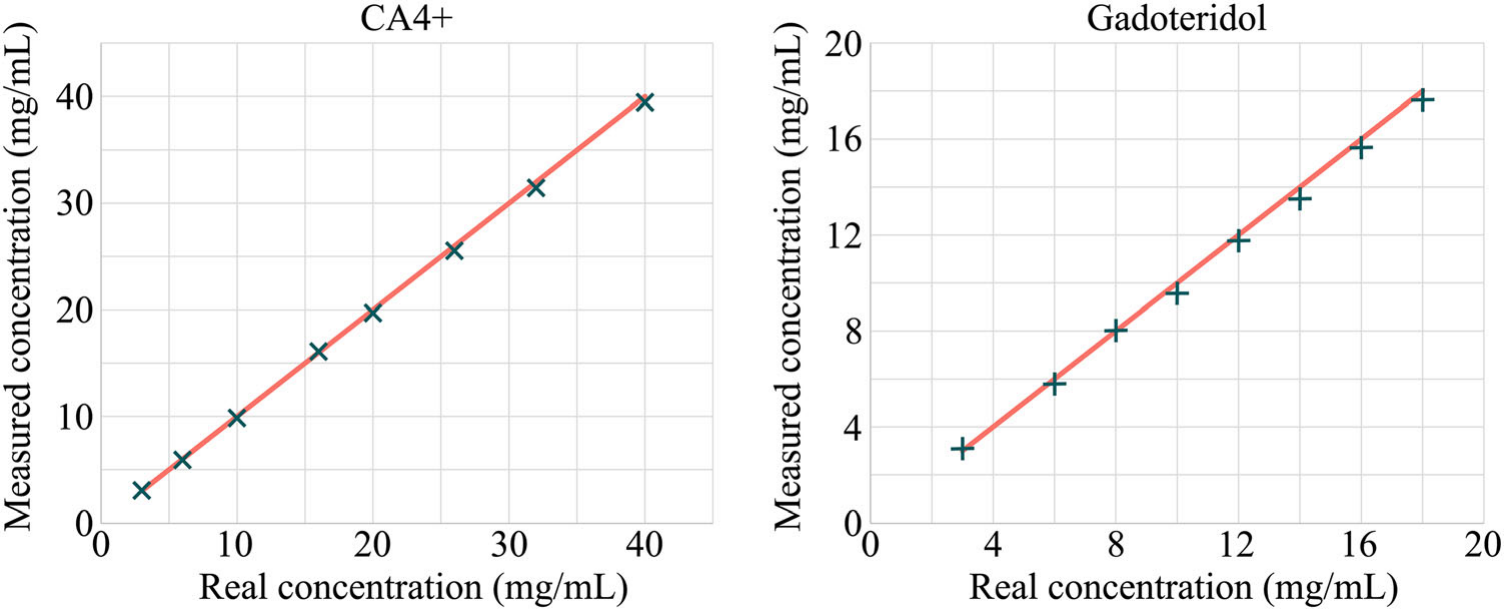
The synchrotron microCT measured CA4+ (×) and gadoteridol (+) concentrations with dual energy technique, and the real concentrations (solid line) within mixture phantoms. The relative mean error in measured concentrations were 1.5 and 2.6% for CA4+ and gadoteridol, respectively.

Before measuring the bovine samples, a set of CA4+ and gadoteridol phantoms with varying concentrations in distilled water were measured with both X-ray energies to determine the mass attenuation coefficients of the contrast agent compounds. The iodine concentrations in CA4+ phantoms were 3, 6, 12, 18, 24, 30, and 36 mg I/mL and gadolinium concentration in gadoteridol phantoms 6, 9, 12, 15, and 18 mg Gd/mL. Based on these calibration measurements, CA4+ and gadoteridol concentrations within the cartilage tissue can be calculated using QDECT. To validate this technique, contrast agent mixture phantoms having iodine/gadolinium concentrations of 3/18, 6/16, 10/14, 16/12, 20/10, 26/8, 32/6, and 40/3 mg/mL were imaged (Fig. 4).

Prior to the contrast agent immersion, non-contrast images of all quarter cartilage samples (*n* = 54) were acquired in the air with 32 and 34 keV X-ray energies. After acquiring the baseline image, the quarter sample pairs obtained from the same patellae were immersed either in dual (*n* = 27) or triple (*n* = 27) contrast agent bath (24 mL, ≥ 100 times the cartilage volume) at + 7 °C. After 2 h, all the samples (*n* = 54) were imaged three times, first with 34 keV X-ray beam, then with 32 keV, and again with 34 keV, to minimize the effect of ongoing diffusion by averaging the two 34 keV images. The acquisition time was approximately 129 s with time difference between the subsequent acquisitions being 331 s on average. Highly optimized algorithm based on Fourier methods was used in image reconstruction.^36^ The measurement set up is explained in more detail in our previous paper by Saukko *et al*.^46^

The absorbed radiation dose on the samples was evaluated by measuring the X-ray flux with calibrated, passivated, and implanted planar silicon (PIPS) diodes.^33^ The measured X-ray flux for 32 keV was 9.8 × 10^10^ photons/mm^2^/s and for 34 keV 1.0 × 10^11^ photons/mm^2^/s. To calculate the absorbed dose, the cartilage samples were modelled as soft tissue (ICRU-44)^56^ according to the X-ray mass energy-absorption coefficient from the NIST database.^14^ The presence of contrast agent was neglected. The absorbed doses were 0.47 Gy for 32 keV and 0.43 Gy for 34 keV.

The articulating surface and bone-cartilage interface at each time point were defined manually using a segmentation software (Seg3D, version 2.2.1, 2015, University of Utah, Salt Lake City, UT, USA) along a protocol described in more detail in our previous paper.^45^ A cylindrical volume of interest (VOI, *d* = 1313 *µ*m, *h* = cartilage thickness) was delineated from the center of the bovine sample using a custom-made MATLAB (R2017b, The MathWorks, Inc., Natick, MA, USA) code. The cracks in the mechanically injured samples were removed by thresholding. Then, the depth-wise X-ray attenuation profiles were calculated, and the profiles obtained from the 34 keV acquisitions were averaged to minimize the error caused by ongoing diffusion. The native X-ray attenuation profiles of the cartilage obtained from the non-contrast-enhanced images were subtracted from the contrast-enhanced profiles. Finally, the depth-wise iodine (CA4+) and gadolinium (gadoteridol) partitions within cartilage were determined in two steps: (1) the mass attenuation coefficients of iodine and gadolinium were determined based on the CA4+ and gadoteridol phantoms; (2) Beer-Lambert law and Bragg’s rule were used to calculate the concentrations of iodine and gadolinium partitions in the cartilage^43^:

1 $$\alpha_{E} = \mu_{{{\text{I}},E}} C_{\text{I}} + \mu_{{{\text{Gd}},E}} C_{\text{Gd }} ,$$

where *α*_*E*_ is the X-ray attenuation with energy *E*, *μ*_I*,E*_ and *μ*_Gd*,E*_ are the mass attenuation coefficients, and *C*_I_ and *C*_Gd_ the concentrations of iodine (I) and gadolinium (Gd), respectively. By applying two energies (32 and 34 keV), the concentrations of iodine and gadolinium at each point can be solved as follows:

2 $$C_{\text{I}} = \frac{{\alpha_{{34\;{\text{keV}}}} \mu_{{{\text{Gd}},32\;{\text{keV}}}} - \alpha_{32} \mu_{{{\text{Gd}},34\;{\text{keV}}}} }}{{\mu_{{{\text{I}},34\;{\text{keV}}}} \mu_{{{\text{Gd}},32\;{\text{keV}}}} - \mu_{{{\text{I}},32\;{\text{keV}}}} \mu_{{{\text{Gd}}, 34\;{\text{keV}}}} }}$$

3 $$C_{\text{Gd}} = \frac{{\alpha_{{32\;{\text{keV}}}} \mu_{{\text{I},34\;{\text{keV}}}} - \alpha_{{34\;{\text{keV}}}} \mu_{{\text{I},32\;{\text{keV}}}} }}{{\mu_{{\text{I},34\;{\text{keV}}}} \mu_{{\text{Gd},32\;{\text{keV}}}} - \mu_{{\text{I},32\;{\text{keV}}}} \mu_{{\text{Gd},34\;{\text{keV}}}} }}.$$

In the analysis, the decrease of the contrast agent concentrations in the surrounding bath due to diffusion was taken into consideration. Moreover, the determined iodine (CA4+) partition profiles were normalized with that of gadolinium (gadoteridol) to reduce the effect of water content and steric hindrance on CA4+ partition and, thereby, to improve the detection of PG content. Further, X-ray attenuation profiles through cartilage depth were divided into superficial (0–10%), middle (10–40%) and deep (40–100%) zones, 0% denoting the articulating surface and 100% the cartilage-bone interface, to compare the contrast agent partition values between the groups (reference, PG-depleted and mechanically injured) and to the corresponding PG distribution (optical density) in each zone.

DD measurements were performed on the second half of the samples to calculate the optical density (OD) by first thawing and subsequently fixing the samples in 10% formalin. Then, ascending series of ethanol and EDTA were used to dehydrate and decalcify the samples, respectively. The samples were embedded in paraffin and cut into 3 *µ*m thick sections. Subsequently, the paraffin was removed, and the samples were stained with Safranin-O which is a cationic dye stoichiometrically binding to the negative fixed charge in cartilage.^19^ To examine the spatial FCD distribution in the cartilage, the optical density (i.e., PG distribution) of the samples were measured using quantitative DD. The equipment included a light microscope (Nikon Microphot-FXA, Nikon Co., Japan) equipped with a monochromatic light source (wavelength 492 ± 8 nm) and a 12-bit CCD camera (ORCA-ER, Hamamatsu Photonics K.K., Japan). The system was calibrated using neutral density filters (Schott, Germany) covering OD range from 0 to 2.6. An average of three sections was used to calculate the depth-wise PG content.

Pearson correlation was used to determine the statistical dependency between the contrast agent partitions and PG distribution within cartilage zones. The significance of enhancement, established by normalization, to CA4+ correlations with PG distribution was tested based on Steiger.^52^ In addition, the significance of the differences in contrast agent partition values between reference samples and conditioned samples were obtained with Wilcoxon signed-rank test. The difference was defined to be significant when *p *< 0.05. The statistical analyses were conducted using SPSS (v. 25.0 SPSS Inc., IBM Company, Armonk, NY, USA).

## Results

**Figure 5:**
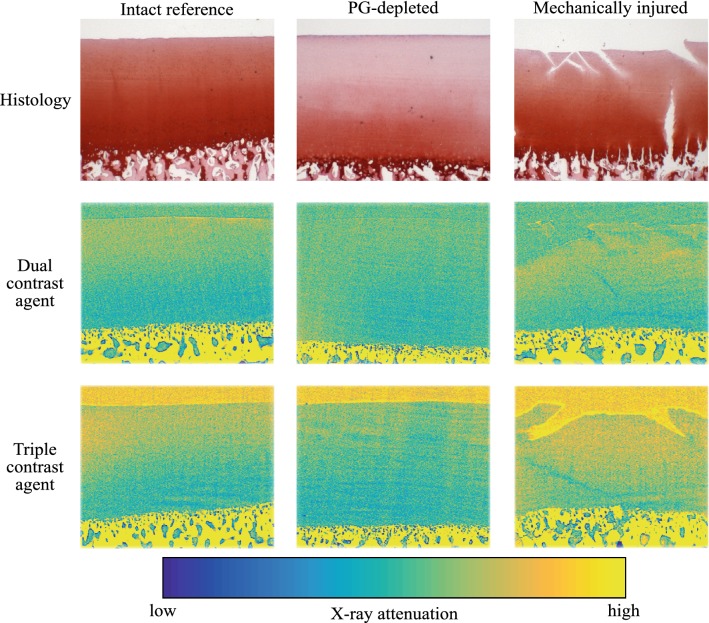
Safranin-O stained histological sections and synchrotron microCT (32 keV) images (average of five consecutive 6.5 *µ*m thick slices) of the intact reference, PG-depleted, and mechanically injured samples imaged with dual and triple contrast agents 2 h after the contrast agent immersion. Articulating surface and cracks are better visualized with the triple contrast agent owing to better contrast induced by bismuth nanoparticles (BiNPs) that, due to their size, are too large to diffuse into cartilage. The enhancement caused by the BiNPs was similar for 34 keV synchrotron microCT images (not shown). CT images were selected to closely match the locations of the histological sections.

The triple contrast agent enhanced the image contrast at the bath-cartilage interface and allowed accurate segmentation of the articulating surfaces at the 2-h time point (Fig. 5). Visual detection of cracks on the cartilage surface of the mechanically injured samples was possible with use of the BiNPs. With the dual contrast agent, the visualization of the bath-cartilage interface and cracks on the cartilage surface was limited. The stability of the BiNPs was measured after mixing the BiNPs, CA4+, and gadoteridol, by determining mean particle diameter at the 2-h time point (193 ± 2 nm; Fig. 3) as well as the 7 and 24-h time points. No change in particle diameter was noted at the 7-h time point, however at the later time point, the diameter of BiNPs slightly increased to 205 ± 4 nm. Even though particle diameter at 24 h was increased, the change was not significant according to the statistical analysis with the One-way ANOVA model (*p *> 0.05) as compared with the original particle diameter. The nanoparticles possessed good colloidal stability in aqueous solution due to the coating with polyethylene glycol (PEG).^40^

**Figure 6:**
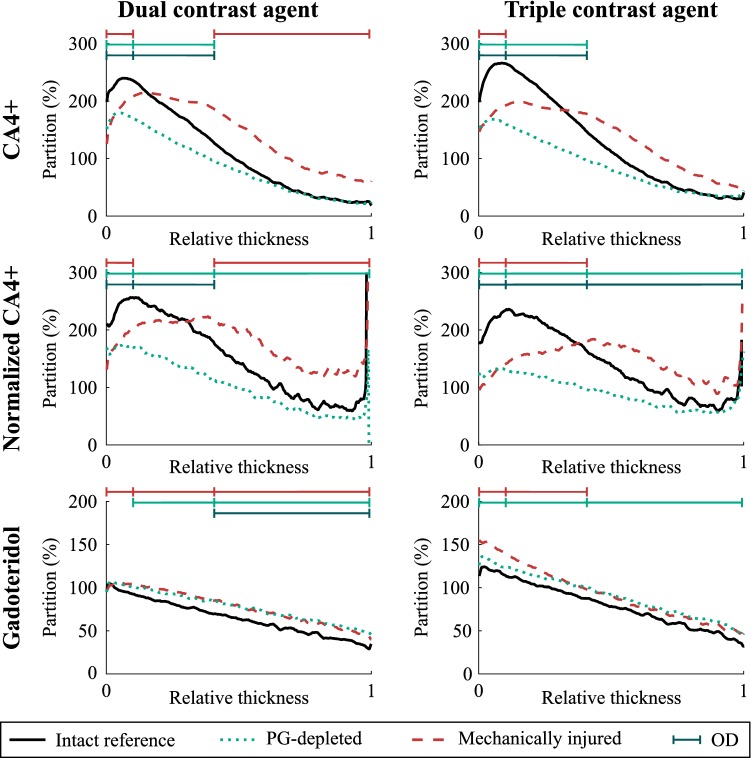
Mean (*n* = 9 for each sample group) depth-wise partitions of CA4+, normalized CA4+, and gadoteridol in cartilage 2 h after the immersion in dual or triple contrast agent. |——| represents the statistically significant (*p *< 0.05) difference between the contrast agent partition in the intact reference and proteoglycan (PG)-depleted (turquoise) or mechanically injured (red) sample and statistically significant (*p *< 0.05) correlation with PG distribution (dark green) within the superficial, middle, and deep zones. Partition is defined as contrast agent concentration in the cartilage divided by the concentration of the immersion bath. 0 denotes the articulating surface and 1 the cartilage-bone interface.

The uptake of CA4+ into intact healthy reference samples was higher as compared with PG-depleted samples, especially at the superficial and middle zones where the difference was significant (Table 1; Fig. 6). A significant difference in CA4+ uptake between intact reference and mechanically injured samples was observed in the superficial and deep zones with the dual contrast agent and in the superficial zone with the triple contrast agent. The normalization of CA4+ partition with that of gadoteridol increased the difference between the intact reference and PG-depleted and mechanically injured samples. In the PG-depleted samples, gadoteridol uptake was significantly higher in all zones for the dual contrast agent while it was greatest in middle and deep zones for the triple contrast agent. In the mechanically injured samples, gadoteridol uptake was greatest in middle and deep zones for the dual agent, and in all zones with triple agent. A statistically significant (*p* < 0.05) difference between the full thickness partitions of the dual and triple contrast agents were observed for CA4+ (reference and mechanically injured samples), normalized CA4 + (mechanically injured samples), and gadoteridol (reference and mechanically injured samples; Table 1). Statistically significant correlations (0.504 < *r* < 0.766, 0.0001<* p* < 0.007) were found between CA4+ partition and PG distribution with both contrast agent mixtures in superficial and middle zones. Gadoteridol partition correlated significantly (− 0.442 < *r* < − 0.428, 0.021<* p* < 0.026) with PG distribution only in the deep zone and whole cartilage thickness with dual contrast agent. The normalization significantly improved the correlation between the CA4+ and PG distribution in the middle zone with both contrast agents. 
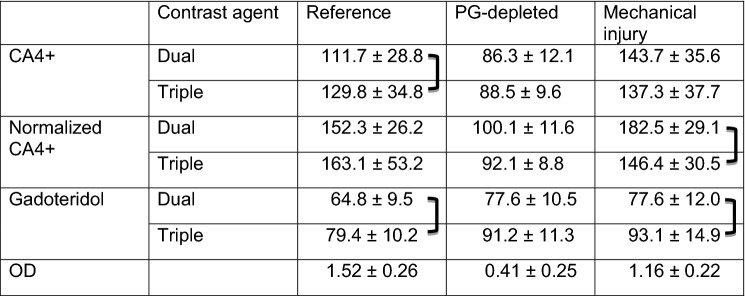

**Table 1:**
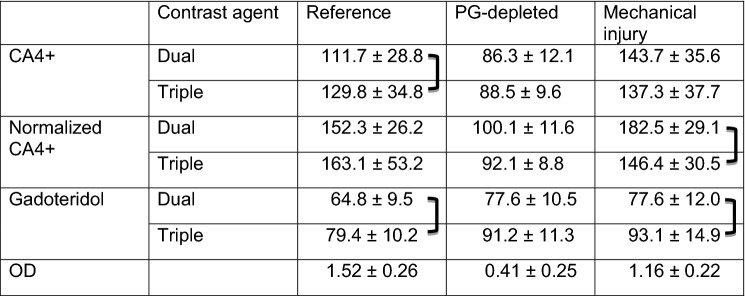
Full thickness contrast agent partitions, standard deviations and optical density (OD) for intact reference, PG-depleted and mechanically injured samples.

Statistically (*p* < 0.05) significant differences between the contrast agent partitions for dual and triple contrast agents are marked with square brackets]

## Discussion

This study evaluates the potential of CECT using the triple contrast agent for assessment of cartilage degeneration and acute injury. We hypothesize that the triple contrast agent will provide a solution for two shortcomings of the current technique. Firstly, simultaneous loss of PGs and decrease in steric hindrance in degenerating cartilage afford opposite effects on CA4+ diffusion, leading to a loss of diagnostic sensitivity at early, clinically feasible, time points. Secondly, contrast at the synovial fluid-cartilage interface is diminished due to contrast agent diffusion into the cartilage, thus limiting detection of this tissue boundary. Accordingly, the triple contrast agent will yield enhanced diagnostic sensitivity for articular cartilage injury and evaluation of cartilage degeneration with lower radiation dose.

Higher CA4+ uptake was seen in reference cartilage samples compared with PG-depleted samples using the dual and triple contrast agents in the superficial and middle zones. During 2 h of diffusion, only a minor portion of the contrast agent molecules have reached the deepest parts of the cartilage and therefore, almost similar CA4+ uptake of contrast agent in intact and PG-depleted samples is observed (Fig. 6). In addition, regardless of the trypsin treatment, some PG molecules remain in the deepest cartilage in the PG-depleted samples. Normalization of CA4+ partition with that of gadoteridol, diminishing the effect of variation in water content on the contrast agent diffusion, increases the difference between the CA4+ partitions of PG-depleted and reference samples. Since nearly all PGs were depleted during the trypsin treatment, the PG-depleted samples bound less CA4+ compared with the intact reference samples with higher PG concentrations in the superficial zone. However, as in this study, imaging was conducted at 2 h after the diffusion afar from the diffusion equilibrium. The contrast agent distribution along the cartilage thickness is not equivalent to that of the PG content (Fig. 5). These results are in agreement with literature.^2^,^11^,^24^,^54^

The uptake of CA4 + into mechanically injured samples, when compared with the intact reference samples, was lower near the articulating surface of the cartilage and higher in deeper with both contrast agent mixtures. This is most probably due to cracks (having exactly 100% partition for CA4+ and gadoteridol) on the cartilage tissue caused by the mechanical impact. Even though the cracks are mostly removed by thresholding, a small margin was left to ensure that no data within cartilage was excluded. Therefore, small parts of the cracks with lower CA4+ partition, when compared with the tissue next to it, are averaged to mean partition in the superficial zone, causing the lower partition than in reference samples at the surface. However, the cracks also facilitate CA4+ diffusion, through the crack walls, into the cartilage, therefore increasing the diffusion surface area, while the diffusion into the reference samples occurs only through the articulating surface.

The uptake of gadoteridol into PG-depleted and mechanically injured samples is higher compared to intact reference samples. This result is a consequence of reduced steric hindrance due to depletion of PGs, disruption of collagen network, and increased water content due to mechanical impact.^9^,^21^ These changes increase the tissue porosity allowing easier penetration of the contrast agent molecules. As the cracks in the mechanically injured samples increase the surface area for diffusion, the uptake of gadoteridol and the gadoteridol partition also increase.

The shapes of the mean (*N* = 9) partition profiles of CA4+ and gadoteridol are similar when using triple or double contrast agents. However, statistically significant (*p* < 0.05) difference between dual and triple contrast agents’ full thickness partitions were found in the reference and mechanically injured samples. The contrast agent partitions for triple contrast agent were slightly higher in the reference and mechanically injured samples (Table 1). These higher partitions of the triple contrast agent’s CA4+ and gadoteridol are most probably due to reconstruction-based increase (in the superficial and middle zones) in attenuation near the articulating surface where the attenuation level drastically changes when the triple contrast agent is used. Moreover, the different halves of the samples were used for dual and triple contrast agent imaging causing minor variation in the cartilage height, due to natural curvatures of the cartilage surfaces leading to a minor difference in the contrast agent partitions. Thus, the significantly lower normalized CA4+ partition for the mechanically injured samples was observed as the slightly lower CA4+ partition was normalized with significantly higher gadoteridol partition. We have ruled out the possibility of BiNPs (particle diameter = 193 nm) to diffuse into cartilage and being responsible for these differing results since BiNPs remains stable for 24 h (Fig. 3) and do not diffuse into cartilage having pore size around 6 nm.^31^,^45^,^49^ As the CA4+ and gadoteridol partitions are slightly higher with the triple contrast agents, BiNPs are not interfering with the diffusion of the other contrast agents. A similar result was observed by Saukko *et al*. with another dual contrast agent (ioxaglate and BiNPs).^45^

In the triple contrast agent, the BiNPs enhanced the contrast between the cartilage tissue and the surrounding contrast agent bath. This enhancement significantly improved the delineation of the articulating surfaces and the detection of surface lesions caused by a mechanical impact compared with dual contrast agent (Fig. 5).

The present triple contrast imaging protocol has three limitations. First, QDECT is based on two image acquisitions conducted simultaneously using two different energies. The energies are selected based on the attenuating elements of the contrast agents, and, the selected energies must straddle both sides of the other elements’ *k*-edge (in this study we chose iodine, 33.2 keV). In the present study, imaging with two energies was performed consecutively with in average 331 s time difference between acquisitions. This difference allows the progression of diffusion within cartilage violating the basic assumption of simultaneous dual energy imaging. However, as imaging was performed first with 34 keV X-ray beam, then with 32 keV, and again with 34 keV, the effect of ongoing diffusion on results was minimized by averaging the 34 keV images. Further, as the image acquisition took approximately 129 s, the obtained attenuation profiles are expected to represent an average over the scan time rather than an exact value at a specific imaging time point. Regardless of this, the results and conclusions are expected to contain no major errors as samples were imaged using the same imaging protocol and as the sample thicknesses were relatively similar. Furthermore, as dual-energy and photon-counting CT scanners become more available, the execution of QDECT technique will become easier and straightforward. Second, the observed minor agglomeration of the BiNPs indicates that BiNPs and the triple contrast agent mixture needs to be prepared just before the immersion/injection, as was done in this study. Third, the technique requires the administration of three contrast agents, and the safety of this combined formulation will need to be addressed. Alone, gadoteridol has been widely used in the clinic, and it is known to possess a very low incidence (1.4%)^13^ of acute adverse reactions. A preliminary study reported the safety of CA4+ on articulating tissues.^53^,^54^ Evaluation of the safety regarding the use of BiNPs is still ongoing. However, bismuth is known to exhibit low toxicity.^37^

To conclude, the triple contrast method enables simultaneous evaluation of PG and water contents, providing information on degenerative changes of articular cartilage. Furthermore, triple contrast agent allows, for the first time, the evaluation of cartilage condition and accurate segmentation of articulating surfaces simultaneously at 2 h post administration. Moreover, taking the advantage of monochromatic X-ray beams with high resolution offered by a synchrotron microCT system, artefacts and limitations related to conventional CT systems are minimized in determining the degenerative state of cartilage. Therefore, this method may enable accurate evaluation of joint health with one image acquisition. As the dual contrast technique with CA4+and gadoteridol successfully images articular cartilage and provides an assessment of proteoglycan and water content in human articular cartilage with a clinical full-body CT,^12^ we are optimistic that the triple contrast method will perform well also in the clinical setup. However, further studies with *ex vivo* knee joints at early diffusion time points and using a full-body CT device are needed to optimize the imaging parameters and imaging time point. These experiments are important in order to reveal the clinical potential of the triple contrast method to detect acute injuries and PTOA related changes in cartilage condition. An extensive time series can be conducted with *ex vivo* setup, as the patient dose is not limiting factor, and with clinical CT system the image acquisition is very fast (< 1 min). Finally, the optimal imaging time point needs to be confirmed with *in vivo* measurements.
